# Reproductive Outcomes Associated with Noise Exposure — A Systematic Review of the Literature

**DOI:** 10.3390/ijerph110807931

**Published:** 2014-08-06

**Authors:** Gordana Ristovska, Helga Elvira Laszlo, Anna L. Hansell

**Affiliations:** 1 Department for Environmental Health, Institute of Public Health of Republic of Macedonia, 50 Divizija No. 6, Skopje 1000, Republic of Macedonia; 2 MRC-PHE Centre for Environment and Health, Imperial College London, London W2 1PG, UK; E-Mails: h.laszlo@ucl.ac.uk (H.L.); a.hansell@imperial.ac.uk (A.H.); 3 Imperial College Healthcare NHS Trust, London W2 1PG, UK

**Keywords:** noise, exposure assessment, birthweight, gestation length, spontaneous abortion, review

## Abstract

*Introduction*: High noise exposure during critical periods in gestation is a potential stressor that may result in increased risk of implantation failure, dysregulation of placentation or decrease of uterine blood flow. This paper systematically reviews published evidence on associations between reproductive outcomes and occupational and environmental noise exposure. *Methods*: The Web of Science, PubMed and Embase electronic databases were searched for papers published between 1970 to June 2014 and via colleagues. We included 14 epidemiological studies related to occupational noise exposure and nine epidemiological studies related to environmental noise exposure. There was some evidence for associations between occupational noise exposure and low birthweight, preterm birth and small for gestational age, either independently or together with other occupational risk factors. Five of six epidemiologic studies, including the two largest studies, found significant associations between lower birthweight and higher noise exposure. There were few studies on other outcomes and study design issues may have led to bias in assessments in some studies. *Conclusions*: There is evidence for associations between noise exposure and adverse reproductive outcomes from animal studies. Few studies in have been conducted in humans but there is some suggestive evidence of adverse associations with environmental noise from both occupational and epidemiological studies, especially for low birthweight.

## 1. Introduction

Noise from the environment, occupational or residential setting is recognized as a stressor agent with sufficient evidence for impacts on hearing impairment, hypertension and ischemic heart disease, annoyance, sleep disturbance, decreased school performance cardiovascular effects and sleep disturbance. For other effects such as changes in the immune system and birth defects, the evidence is limited [1,2,3]. There is no doubt that noise along with a variety of other occupational and environmental conditions acts as general stressor on the mother inducing a variety of physiological and psychological changes that may affect pregnancy [4,5].

Sufficient published evidence supports the notion that stress triggers the release of neurohormones by the hypothalamus-pituitary-adrenal (HPA) axis, and subsequently the activation of the HPA axis stimulates up-regulation of key stress hormones such as corticotropin-releasing hormone (CRH), adrenocorticotropic hormone (ACTH) and glucocorticoids (GCs) [6]. Neurohormonal responses to stress also include an activation of the sympathetic nervous system with successive increased secretion of catecholamines, a phenomenon that has received much less attention than the stress-triggered activation of the HPA axis [7]. Research findings suggest that neurotrophin nerve growth factor (NGF) has a role of a critical arbitrator of stress responses. Circulating levels of NGF undergo considerable modification during a stress challenge and promote ‘cross-talk’ between neuronal and immune cells, ultimately skewing the immune response towards inflammation [8]. The neuropeptide substance P (SP) is another major mediator of the systemic stress response and SP can be considered a pivotal stress-related neuropeptide, triggering distortion of the immune response towards inflammation [6].

Investigators in experimental studies have exposed animals to different noise types and intensity, with the aim to simulate environmental conditions for noise exposure in humans. In the course of studying the mechanism for development of reproductive outcomes, plasma levels of stress hormones like corticosterone [9], norepinephrine (NE), epinephrine (EPI), uterine NE [10], adrenocorticitropic hormone (ACTH) plasma levels [11] have been investigated. Nawrot *et al.* didn’t find significant noise-related changes in plasma corticosterone levels, but did find decreased mean foetal weight and increased embryo and foetal mortality in studies in mice [9]. Cook *et al*. found significant elevation of plasma EPI and NE levels in noise-exposed mice, significantly decreased foetal weights and decreased maternal weight gain [10]. Bailey *et al*. found elevation of ACTH plasma level in noise-exposed guineapigs [11]. Kimmel *et al*. [12] observed significantly increased resorption rates and decreased number of live fetuses per litter in each of exposed groups of animal, but no teratogenic effects were noticed among the exposed mice. Murata *et al*. [13] found significant differences in malformed fetuses between the control and group exposed to noise on day seven of pregnancy. Rasmusen *et al*. [14] found significant correlation between number of stillborn pups and noise exposure, even at 70 dBA for 1 h. Sato *et al*. [15] found significant decrease of birth rates in female rats exposed to noise during copulation and pregnancy, and number of offspring by group decreased in exposed female rats through copulation and pregnancy.

Meyer *et al*. reviewed six epidemiological studies on reproductive outcomes and concluded that effects of noise were equivocal. The authors commented on the ecological nature of the studies, misclassification bias in exposure assessment and inadequacies in addressing the impact of confounding factors [4]. Hepper and Shahidullah reviewed eight epidemiological studies in a report published in 1994, including five of the six studies reviewed by Meyer *et al*. [4]. They comment that several of the studies reported some evidence of an association, but overall there was no conclusive evidence for an association between reproductive outcomes like low birthweight, prematurity, congenital malformations and noise exposure [5]. Hohmann *et al*. performed a systematic review on noise exposure and birth outcomes published in 2013 [16], which included 12 studies, nine studies with information on occupational noise and three with information on environmental noise, two of which had been included in the previous reviews [4,5]. Hohmann *et al*. [16] concluded that chronic occupational exposure of pregnant women did not seem to be associated with birthweight, preterm birth and fetal growth, while studies of environmental noise were inconclusive.

Low birthweight (LBW) is defined as an infant weight of less than 2500 g irrespective of gestational age. LBW infants are either those who experience normal growth, but are born too early (preterm) or those who are born pre-term or full term, but have inadequate fetal growth (intrauterine growth retardation) [17]. The World Health Organization defines preterm birth as a gestational age at birth of less than 37 completed gestational weeks [18]. Low birthweight and preterm births are recognized as a major public health problem by both, clinicians and researchers because they are the leading cause of infant mortality and also contribute to substantial neurological, cognitive, pulmonary and ophthalmologic morbidity [19]. Caring for preterm infants also incurs large health care expenditures. Mild- and moderate-preterm birth infants are at high relative risk for death during infancy and are responsible for an important fraction of infant deaths [20]. Reduction to one third of the proportion of infants with LBW is one of the seven major goals of the current decade of the “A world fit for children”, program of the United Nations [19].

The aim of this paper is to undertake a systematic review of published evidence investigating reproductive outcomes like low birthweight (LBW), preterm births (PB), spontaneous abortions, congenital malformations in humans related to occupational and environmental noise exposure and to give directions and recommendations for further research on reproductive outcomes.

## 2. Material and Methods

### 2.1. Search Strategy

A systematic search was conducted on noise and reproductive outcomes. Web of Science, PubMed and Embase electronic databases were searched for papers published between 1970 to June 2014. Studies were also screened in the reference list of relevant reviews/articles. In addition, hand searching was used for acoustical conference proceedings (Internoise 2000–2002, 2004–2005, 2007–2008, 2010). No language restriction was applied. The following search terms were used: noise AND health AND perinatal OR prenatal OR labour OR birth OR malformation OR gestation OR preterm OR foetus OR pregnancy.

### 2.2. Study Selection

Five inclusion criteria were defined. The paper was included if: (a) it described noise exposure (objective/subjective assessment), (b) the source of noise was either environmental (road traffic, railway or aircraft noise) or occupational, (c) the study investigated the following reproductive outcomes: birthweight/gestation length/preterm birth/prematurity/reproductive health/congenital malformation/foetal growth retardation/small-for-gestational-age infant/spontaneous abortion, (d) the above health outcomes occurred during pregnancy or delivery up to 4 weeks after birth and (e) the paper examined a direct relationship between the above health outcomes and noise exposure. Studies investigating health outcomes other than those listed in the inclusion criteria such as pre-eclampsia, hearing development, male reproductive function, or health outcomes that occurred after the 4th week of birth were not included in this review. Case studies or case reports, studies containing no original research and studies investigating different noise source such as neonatal intensive care unit (NICU) noise were excluded as were studies looking at distance from road only without other assessment of noise exposures.

The database search yielded 2356 references (last access on 7 July 2014, 598 records in Web of Science, 1489 records in PubMed and 269 records in Embase). Abstracts of potentially eligible studies were read and judged against inclusion criteria by one reviewer (HL). Sixty potential papers were then retrieved and read in full by two reviewers (GR, HL). We could not find the full paper for five articles and three references were books or Ph.D. theses; they were not available and we decided to exclude them from the list (GR, HL). No conference proceedings matched the inclusion criteria. After carefully reading full papers, we found that 12 of them were reviews, 11 papers had different outcomes that defined in inclusion criteria, two papers had results already published in other articles, two papers had very low quality assessment score, and two papers were without noise exposure assessment. Discussion about exclusion of these papers was performed with the third review author (AH). Finally we agreed on 23 papers to be included in this review.

### 2.3. Data Extraction and Quality Assessment

We developed data extraction sheets which contained the following characteristics: author, year of publication, country, study design, sample size, exposure assessment (indicators and range of exposure), outcome, confounding factors, effect size and quality score. Two reviewers (GR and HL) independently worked on data extraction and quality assessment of the studies and agreement was reached via consensus. For the purpose of this review we developed our own method for quality assessment (QA) of the epidemiological studies, using some criteria from the Newcastle–Ottawa quality assessment scale for case control and cohort studies [21]. Final criteria for QA of the studies were:

- Publication type (0 = not peer reviewed, 1 = peer reviewed article),
- Study design (1 = ecological, 2 = case control or cohort study, 3 = RCT, 0 = other),
- Noise exposure assessment (1 = subjective assessment by the mother, 2 = expert assessment e.g., conducted by an industrial hygienist, 3 = objective assessment with noise measurements),
- Assessment of reproductive outcomes (1 = subjective assessment by report of mother, 2 = objective e.g., from medical records),
- Confounding factors (0 = no confounding factors considered, 1 = confounding factors considered but some key confounders omitted, 3 = careful consideration of confounders),
- Statistics (0 = flaws in or inappropriate statistical testing or interpretation of statistical tests that may have affected results, 1 = appropriate statistical testing and interpretation of tests),
- Bias (0 = other study design or conduct issues that may have led to bias, 1 = no other serious study flaws).

For this scale, the maximum total score can be 14. Where the total score of the study ≥10, this was assessed as a study with strong evidence, a study with score from 6–9 was assessed as moderate evidence, and score ≤ 5 was assessed as insufficient evidence. Differences in the review process were solved with the opinion from the third reviewer (AH).

## 3. Results and Discussion

We selected 14 epidemiological studies related to occupational noise exposure and 9 epidemiological studies related to environmental noise exposure for this review.

### 3.1. Evidence from Occupational Studies

Table 1 summarizes the characteristics of epidemiological studies performed to investigate independent effects of noise exposure on reproductive outcomes in occupational environment. We identified 14 studies for this review eligible for this review, two of them were surveys [22,23], ten case control studies [24,25,26,27,28,29,30,31,32,33] and two prospective studies [34,35].

The following ten studies were assessed as studies with strong evidence (quality scores ≥10); findings were not consistent across these studies. Nurminen and Kurppa (1989) performed a case-control study to examine threatened abortion and they found significant risk in noise exposed women together with shift work (RR = 2.1 95% CI 1.0–4.6). Noise exposure was blindly assessed from a description of the mother’s workday by two industrial hygienists. Women with an estimated level of noise of Leq 8 h > 80 dB were considered exposed [25]. Zhan *et al*. in their study performed in China, used objective noise exposure assessment and divided exposed groups into subgroups according to noise exposure levels. The subgroup exposed to noise level from 95–99 dBA showed significant risk for LBW (OR = 3.9 95% CI 2.3–6.7) and for spontaneous abortion (OR = 2.2 95% CI 1.3–3.8) compared with those <75 dBA. The subgroup exposed to noise level from 100–104 dBA showed significant risk for LBW (OR = 3.7 95% CI 3.2–6.2) and spontaneous abortion (OR = 3.0 95% CI 1.8–4.9) [27]. Another study where noise exposure assessment was subjectively evaluated, did not show a significant risk for preterm birth, threatened abortion and congenital malformations [28]. Luke *et al.* performed a large case control study in nurses in the USA and found significant risk for preterm births (OR = 2, *p* = 0.005, no confidence intervals reported) [29], but another study with strong evidence performed in Europe did not find significant risk for preterm birth where other occupational conditions were carefully considered [32]. Hruba *et al*. [30] conducted a case control study investigating intrauterine growth retardation in newborns in the Czech Republic and found significant risk in noise exposed women, according to the women’s subjective assessment of noise exposure.

**Table 1:** Summary of epidemiological studies of occupational noise exposure and reproductive outcomes (ordered by year of publication).

Author, Year	Country	Study Design	Sample Size	Exposure Assessment	Outcome	Confounding Factors	Effect Size for Noise*	Quality Score
Mcdonald *et al*., 1986 [22]	Canada	Survey	56,012 women	Subjective	Spontaneous abortion, (before 28th week of pregnancy)	Maternal age, education, smoking, parity, obstetric history, occupationla factors	O/E = 1.17; *p* < 0.05 in office work O/E = 1.48; *p* < 0.05 in sales O/E = 1.40; *p* < 0.01 in service	9
McDonald *et al*., 1988 [23]	Canada	Survey	22,761 live newborns	Subjective	LBW Gestation length (<37 weeks)	Maternal age, education, ethnic group, gravidity, smoking, alcohol intake	O/E = 1.49 ( *p* < 0.01) for health sector O/E = 1.20 (*p* < 0.05) for manufacturing sector *p* = 0.02 Nonsignificant O/E	9
Hartikainen-Sorri *et al*., 1988 [24]	Finland	Case-control study	284 cases and 299 controls	Subjective	Preterm birth LBW	Socioeconomic factors, type of the work, occupational coexposures, smoking	RR = 0.7 (95% CI 0.1–3.4) RR = 2.4 (95% CI 0.2–20.2)	9
Nurminen *et al.*, 1989 [25]	Finland	Case-control study	1475 subjects	Subjective, Three groups exposed to Leq 80 dBA, 85 dBA and 90 dBA	Threatened abortion SGA	Maternal age and weight, parity, smoking, alcohol intake	RR = 2.1 (95% CI 1.0–4.6) with shift work RR = 1.4 (95% CI 0.8–2.6)	12
Kurppa *et al*., 1989 [26]	Finland	Case-control study	402 cases and 440 controls	Subjective, three groups exposed to Leq 80 dBA, 85dBA and 90dBA	Structural malformations	Socioeconomic factors, obstetric history, type of the work, occupational coexposures	OR=0.9 (95% CI 0.7–1.0) According to mother’s evaluation OR=1.7 (95% CI 0.7-4.1) According to industrial hygienist evaluation	12
Zhan *et al*., 1991 [27]	China	Case-control study	978 cases and 402 controls	Objective, Three groups exposed to Leq = 85–94 dBA, 95–99 dBA, 100–104 dBA	Spontaneous abortion LBW	Maternal age, occupational factors	95–99 dBA OR = 2.2 (95% CI 1.3–3.8) 100–104 dBA OR = 3 (95% CI 1.8–4.9) 95–99 dBA OR = 3.9 (95% CI 2.3–6.7) 100–104 dBA OR = 3.7 (95% CI 3.2–6.2)	13
Zhang *et al*., 1992 [28]	China	Case-control study	1875 cases and 1875 controls	Subjective	Small for gestational age Preterm birth Threatened abortion Congenital malformations	Gender, mother’s age, plurality, parity, coexposures to radiation, chemicals, pesticides	OR = 0.8 (95% CI 0.5–1.5) OR = 1.1 (95% CI 0.7–1.9) OR = 1.1 (95% CI 0.5–2.1) OR = 1.3 (95% CI 0.8–2.2)	11
Hartikainen *et al*., 1994 [34]	Finland	Prospective study	111 exposed women and 181 unexposed women	Objective, cut off point for exposure Leq 8 h > 90 dBA	Low birthweight (LBW)	Socioeconomic factors, age, parity, marital status, smoking alcohol, type of the work	Decline in absolute birthweight, (mean 3304 g (SD 585) for the exposed *vs*. mean 3622 g SD 548) for the unexposed.	9
Luke *et al.*, 1995 [29]	USA	Case-control study	210 cases and 1260 controls	Subjective	Preterm births (<37 weeks)	Maternal age, race, education, marital status, smoking, occupational fatigue score	OR = 2 *p* = 0.005	10
Hrubá *et al*., 1999 [30]	Czech Republic	Case-control study	3897	Subjective	Intauterine growth retardation (IUGR)	Maternal age, education, smoking, shiftwork, standing, lifting, exposure to chemicals	OR = 1.9 CI not available *p* = 0.03	11
Chen *et al*., 2000 [31]	China	Case-control study		Subjective	LBW	Maternal age, education, occupation, smoking, alcohol intake, occupational coexposures	Estimated change in birthweight 14 *p* = 0.69	10
Saurel-Cubizolles *et al*., 2004 [32]	European study	Case-control study	5145 preterm and 7911 term births,	Subjective	Preterm birth (<37 weeks)	Maternal age, education, marital status, obstetric history, occupation, working conditions, occupational coexposures	OR = 0.99 95% CI =0.9–1.1	10
Magann *et al*., 2005 [35]	USA	Prospective study	814 low risk healthy women	Objective, LAeq 8 h, cut off point for exposure was 85 dBA	Preterm birth Preterm labor IUGR Perinatal death	Maternal age, weight, education, family factors, occupational coexposures	OR = 0.8 (95% CI 0.1–2.9) OR = 2.5 (95% CI 0.6–7.5) OR = 0.2 (95% CI 0.02–0.5) OR = 0.9 (95% CI 0.2–2.7)	13
Croteau *et al*., 2006 [33]	Canada	Case-control study	276 cases 640 controls	Subjective	Small for gestational age (SGA)	Maternal age, weight, education, family factors, obstetric history, smoking, alcohol intake, occupational coexposures	OR = 1.2 (95% CI 1.0–1.5)	11

Notes: OR (Odds Ratio), CI (Confidence Intervals), O/E (Observed/Expected); * results are reported to two decimal points except where original paper uses one decimal point.

A large follow up study in petrochemical industry investigated effects on birthweight due to noise exposure and other chemical and physical occupational factors in women but didn’t find independent influence of noise on birthweight in multivariate models [31].

The effects of occupational factors on pregnancy were analyzed in a large prospective study with low-risk healthy working women [33]. The independent effect of noise exposure on reproductive outcomes didn’t reach statistical significance, but a multivariate analysis of non-exposed compared with exposed women found an effect of standing on preterm labor (OR 1.80, 95% CI 1.05–3.16) and preterm birth (OR 1.69, 95% CI 1.03–2.80) and showed a trend towards an effect of noise exposure on preterm labor (OR 1.76, 95% CI 0.78–3.39) after controlling for other exposures.

Croteau *et al*. [33] performed a case–control study to evaluate whether some occupational conditions during pregnancy increase the risk of delivering a small-for gestational-age (SGA) infant and whether taking measures to eliminate these conditions decreased that risk. The risk of having an SGA infant increased with an irregular or shiftwork schedule alone and with a cumulative index of the occupational condition like noise exposure. When the noise exposure was not eliminated, the risk increased OR = 1.2 (1.0–1.5), but prevention of noise exposure before 24th week of pregnancy brought the risks close to those of unexposed women (OR = 0.9, 95% CI 0.6–1.4).

Kurppa *et al*. [26] tested the hypothesis that occupational noise exposure during pregnancy was teratogenic. They obtained data from Finnish Register of Congenital Malformations supplemented by special interviews on the mothers' work conditions. This included 1475 Finnish mothers who had given birth to a malformed child (orofacial cleft or structural defect of the central nervous system, skeleton, or heart and great vessels) and 1475 reference mothers. Occupational hygiene assessment according to expert opinion of industrial hygienist indicated that 102 case mothers and 103 referents had been exposed in the first trimester to a noise level of Leq (8 h) > 80 dB, the overall OR being 1.0 (95 % CI 0.7–1.3). 

Four studies were assessed as providing moderate evidence. Within a large survey of pregnancies in Montreal, McDonald *et al*. [23] studied the frequency of LBW (<2500 g) and GL (<37 weeks) of 22,761 single live births in relation to maternal employment, taking into account of 11 non-occupational confounding factors. Noise exposure was associated with LBW, only in the women who worked in health and manufacturing sectors. Gestation length was not associated with noise exposure. The authors also investigated spontaneous abortion [22] and found significant risks only in women who worked in sales (OR = 1.48) and service (OR = 1.40) and office work (OR = 1.17), but the association with noise was not statistically significant when ranking by job demands rather than occupational groupings (while physical effort was consistently statistically significant). Subjective assessment was used to determine noise exposure, while other occupational conditions and individual factors were controlled. Hartikainen-Sorri *et al*. [24] performed a case-control study of 299 women with LBW babies (<25th centile) and 284 women with preterm birth and matched controls. The study did not find a significant association with occupational noise, but they had a very small sample (26 subjects) of highly noise-exposed women, Leq(A) 8 h (≥81 dB). Hartikainen *et al*. [34] performed a prospective cohort study with objective noise exposure assessment where the cut-off point for noise exposure was LAeq > 78 dBA. They found birthweight was on average 200–300 g lower in the group exposed to >90 dBA (Leq, 8 h) with mean 3304 (SD 585) g for the exposed *vs.* mean 3622 (SD 548) g for the unexposed.

The main limitation of epidemiological studies regarding occupational noise exposure was subjective noise exposure assessment, *i.e.*, according to the mother’s opinion of whether she considered that she was exposed to occupational noise during pregnancy. Subjective evaluation of noise exposure is important because it is likely to be in close relation to the stress response of each person, but we also need accurate noise exposure assessment, which can be readily obtained in occupational settings. The other option for subjective assessment is according to the judgment of industrial hygienist, e.g., did he/she consider that specific occupational settings had a higher level of noise exposure (such as above 85 dBA). There is big possibility for exposure bias in performing such studies. Only three of the 14 occupational studies—two prospective studies [34,35] and one case control study [27] used objective exposure assessment methods, with noise measurements. In the two prospective studies using objectively assessed noise exposure, which also assessed influence of other occupational factors, the independent effect of noise didn’t reach statistical significance [34,35]. In the case-control study, the two subgroups exposed to (objectively assessed) noise above 95 dBA (*i.e.*, very high noise levels) showed significant risk for LBW and spontaneous abortion [27].

### 3.2. Evidence from Epidemiological Studies

Table 2 summarizes characteristics about the nine epidemiological studies included in the review investigating environmental noise exposure and reproductive outcomes. Four of these were case-control studies [36,37,38,39], two were surveys [40,41], one was a cross-sectional study [42], one was a prospective study [43] and one was population based cohort study [44]. Objective noise exposure assessment was used in eight of these studies. Most of the studies examined aircraft noise exposure and its influence on LBW and some of them investigated dose-response relationships between noise exposure and low birthweight. According to the quality assessment score, six studies were assessed as providing strong evidence and three studies provided a moderate evidence score. Those scored as providing strong evidence are discussed first.

Schell in 1981 [42] examined the association between maternal exposure to aircraft noise and birthweight or gestation length in a relatively small study involving 115 infants but where noise exposure assessment was performed with measurements of SEL during airplane take-off, ranging from 75–100 dBA. The birthweight and other data were collected through personal interviews with the mothers. They found a significant negative partial correlation between noise exposure and gestation length in female infants, controlling for maternal age, smoking, parity, socioeconomic status, and parental height and weight (r = −0.49, *p* < 0.001). Noise exposure also showed a slightly negative correlation with male birthweight and gestation length and with female birthweight; however, these correlations were not statistically significant.

**Table 2:** Summary of epidemiological studies on environmental noise exposure and reproductive outcomes (ordered by year of publication).

Author, Year	Country	Study Design	Sample Size	Exposure Assessment	Outcome	Confounfing Factors	Effect Size	Quality Score
Ando and Hattori, 1973 [36]	Japan	Case-control study	713	Objective assessment, aircraft noise, ECPNL (dB)	LBW (<2500 g)	Gender, maternal age, occupation, parity	Higher rate of LBW in noisy area above 75 dBA	8
Ando and Hattori, 1977 [37]	Japan	Case-control study	343 cases and 112 controls	Objective assessment, aircraft noise, 75–95 dBA noise exposure	Human placental lactogen (HPL) levels in maternal serum Birthweight	No confounders	Significant lower HPL level in noise exposed women after 32nd week of pregnancy Significant correlation between birthweight and lower HPL level (≤4 mg/mL)	9
Edmonds *et al.*, 1979 [40]	USA	Survey	1745 birth defects	Objective assessment, aircraft noise, high noise level exposure above 65dBA Ldn	17 categories of birth defects	Socioeconomic status, race	Non significant differences in rates of birth defects in exposed and nonexposed groups	10
Knipschild *et al*., 1981 [38]	Netherlands	Case-control study	1840	Objective assessment, aircraft noise, 3 subgroups Ldn < 65 dBA, Ldn 65–70 dBA, Ldn > 70 dBA	LBW	Gender, parental income	18% LBW in low noise exposed group, 24% LBW in high noise exposed group, 29% in noise exposed above 70 dBA Dose response relationship between aircraft noise and LBW	8
Schell, 1981 [42]	USA	Cross- sectional study	115	Objective assessment, aircraft noise, SEL = 75–100 dBA	Birthweight Gestation length	Maternal age, obstetric history, parental weight and height, education, smoking, family income	r = −0.04 *p* = 0.76 males r = −0.22 *p* = 0.014 females r = −0.18 *p* = 0.16 males r = −0.38 *p* = 0.008 females	11
Wu *et al*., 1996 [43]	Taiwan	Prospective study	200	Objective and subjective assessment, Leq24 hours	LBW	Maternal age, weight gain, gender and gestational age, socioeconomic status	Non-significant correlation between noise exposure and LBW	13
Matsui *et al*., 2003 [41]	Japan	Survey	160,460 births	Objective assessment, aircraft noise, WECPNL (dB) Control group <75 dBA Exposed subgroups 75–80 dBA, 81–85 dBA, >85 dBA	LBW (<2500 g) Preterm birth (<37 weeks)	Gender, maternal age, socieocnomic status, live birth order	OR = 1.3 (95% CI = 1.1–1.4), *p* = 0.0001 in the highest level of exposure OR = 1.25 (95% CI = 1.1–1.4), *p* = 0.0018 in the highest level of exposure	10
Wang *et al*., 2011 [39]	China	Case-control study	60 cases and 120 controls	Subjective assessment, residential noise exposure	Recurrent spontaneous abortion	Individual and family factors, other environmental factors	OR = 5.39 95% CI 1.03–28.20 Noise exposure over 6 hours increased the risk for spontaneous abortion	11
Gehring *et al.*, 2014 [44]	Canada	Retrospective study of birth records population based cohort study	68,238 births	Objective, noise modeling	Preterm birth LBW Small for gestational age	Gender, ethnicity, parity, family income, education, smoking, air pollution	OR = 1.03 (95% CI 0.99–1.07) OR = 1,11 (95% CI 1.02–1.19) OR = 1.10 (95% CI 1.06–1.13)	13

Notes: OR (Odds Ratio), CI (Confidence Intervals), ECPNL (Equivalent Continuous Perceived Noise Level), SEL (Sound Exposure Level), r (correlation coefficient), WECPNL (Weighted Equivalent Continuous Perceived Noise Level).

Wu *et al*. [43] performed most detailed exposure assessment, using three different methods: personal noise dosimeter performing noise measurements for 24 h; traffic noise exposure assessment using noise maps for residential areas of the participants; and self evaluation of the habits for listening to loud music and using personal listening devices during pregnancy. The mean value and standard deviation of individual exposure Leq 24 h was 67.9 dBA, (52.4 dBA–86.8 dBA). A well-characterized cohort of 200 pregnant women was followed during pregnancy and data for birthweight were obtained from medical records. The authors didn’t find any statistically significant associations between personal noise exposure measured and low birthweight (*p* = 0.24), between traffic noise exposure indicating by the distance between the home and main streets and low birthweight (*p* = 0.17), between using personal listening musical devices during pregnancy and infant birthweight (*p* = 0.34).

Matsui *et al*. [41] conducted a large study of 160,460 birth records from 1974 to 1993 and found a very highly statistically significant dose-response relationship between LBW and aircraft noise exposure. The adjusted OR for LBW was 1.3 and for preterm birth OR = 1.2 in highest noise exposure group. The authors adjusted for confounding factors like gender of the infant, maternal age, birth order, occupation of householder, but they didn’t adjust for smoking habit of mothers, which may have resulted in some residual confounding.

Only one study used subjective noise exposure assessment, using the mother’s opinion about whether she felt she was living in noisy environment. Wang *et al*. [39] investigated recurrent spontaneous abortions in a case-control study, performed in China and they found noise exposures of more than 6 hours in a residential area was associated with recurrent spontaneous abortion (OR = 5.3 95% CI 1.0–28.2). This outcome was adjusted for education, infections of the reproductive tract and husband’s alcohol drinking, but the study had serious limitations with great possibility of exposure measurement bias as this was assessed subjectively and thus may relate to general stress levels or other environmental factors rather than noise level itself. 

Gering *et al*. [44] linked nearly 70,000 administrative birth records in Vancouver, Canada to modeled residential road traffic and all transportation noise exposure. After controlling for various factors including income and education, a statistically significant negative association was seen between road traffic exposure and term birthweight with mean difference = −19 g (95% CI = −23 to −15) per 6 dBA. Results were robust to adjustment for air pollution exposure. Similar sized negative associations were also seen with combined road, aircraft and railway noise, but the latter two sources were only a minor contribution to community noise. The study also found significant risk for small for gestational age OR= 1.10 (1.06–1.13), but not on preterm or very preterm birth. In joint noise-air pollution models, there were independent effects of noise and air pollution exposure on small for gestational age.

Edmonds *et al*. [40] investigated the incidence of birth defects in two groups of infants whose mothers lived around Atlanta airport, exposed to Ldn above 65 dB and below 65 dB, but did not find a significant association.

The three studies whose quality was assessed as moderate investigated aircraft noise. In a case-control study, Ando and Hattori [36] found increased incidence of low birthweight in mothers who were exposed to aircraft noise. Noise exposure assessment was performed using the indicator ECPNL (dB), divided into five subgroups of exposure in range of 74–90 dBA. The relative low birthweight rate was 3% lower for the three year period in the noisy area (above 75 dBA) compared to the neighboring quiet cities, not exposed to jet aircraft flights. Following jet planes starting to fly regularly, relative low birthweight rate increased and it was over 5% for both males and females. Ando and Hattori also [37] investigated the levels of human placental lactogen HPL in the serum of mothers both subjected to and not subjected to aircraft noise. HPL is also known as human chorionic somatomamotrophin with biological properties known as growth stimulation and lactogenic activity and impacts on HPL may be a potential mechanism for growth inhibition. The HPL levels in the quiet reference area and in the noise area were similar before the 29th week of pregnancy. However, the HPL levels of noise exposed subjects tended to be lower than those in the reference area after the 30th week of pregnancy and the difference became significant after the 36th week of pregnancy. The percentage of mothers with HPL levels that could be potentially dangerous for the fetus tended to be higher in the noise exposed group. The lower HPL levels were associated with lower birthweight for infants from mothers exposed to noise.

Knipschild *et al*. in a study published in 1981 [38] compared the birthweight of 498 infants whose mother lived in a noisy area near the Amsterdam airport with that of 404 infants from less noisy areas. Mothers exposed to Ldn < 65 dBA had 18% infants with LBW, mothers exposed to Ldn 65–70 dBA had 23% infants with LBW and mothers exposed to Ldn > 65 dBA had 29% infants with LBW. These findings were then adjusted for parent’s income, mother’s age, birth order, twinship and sex of the infant (but not for mother’s smoking). After adjustment for family income, the association was present only among female infants.

Only the abstract could be located for a study by Jones and Tauscher [45] so it could not be fully assessed and is not included in Table 2. The authors investigated congenital anomalies near Los Angeles airport and found greater incidence of all birth defects among black infants in areas where the noise exposure was >90 dBA compared to those who were not exposed to aircraft noise. However, when the incidence of anencepahaly and spina bifida was examined alone in white infants an increased incidence was noted among infants whose mothers lived near the airport.

### 3.3. Potential Confounding Factors

Occupational studies included in this review found that several occupational and non-occupational factors influenced reproductive outcomes. Occupational factors involved were standing, lifting and exposure to chemicals—usually persons who were exposed to noise were exposed to other occupational factors. Important non-occupational factors were mother’s age, mothers weight and height, mother’s weight gain during pregnancy, smoking, education, race and socioeconomic status. Gravidity and parity, and chronic diseases of the mother were also important factors for examination of spontaneous abortion or preterm labour.

The impact of confounding factors with strong evidence that they influence on birthweight was inadequately addressed in most of the environmental noise studies. Included among these factors are maternal age, parity, gravidity history, smoking and socioeconomic status. Some of this information (e.g., age, parity) probably would have been readily available on the vital records and other sources of data used in these studies, yet it was not utilised. 

### 3.4. Summarising the Evidence from Occupational and Epidemiological Studies

*LBW* was investigated in five occupational studies [23,24,27,28,31] but only two used objective exposure assessment and these found significant risk for noise level above 95 dBA [27] and decline of mean birthweight from mothers exposed to noise above 90 dBA [34]. The other studies used subjective evaluation of noise exposure assessment and adjustment for other occupational factors, and they didn’t find significant associations. One study found significant risks of noise only for mothers who work in health and manufacturing sector [23]. LBW or birthweight was also investigated in six epidemiological studies [36,38,39,41,42,43,44]. The two largest studies found associations with LBW. One study from Japan [41] found significant risk for LBW for mothers exposed to aircraft noise above 85dBA and another large population base cohort study from Canada [44] that found adverse effects of road traffic noise exposure and for all transportation noise associated with term birthweight and term very low birthweight. The noise effect on term birthweight was largely unchanged after adjustment for air pollution [44]. Two smaller studies with lower quality scores also saw higher risk of LBW with higher noise exposure [36,38]. A further two studies investigated correlations not risks, finding associations with birthweight in female but not male babies [42] or no association with LBW [43]. There is therefore evidence supportive of associations between LBW and noise exposure including from the better designed and larger occupational and epidemiologic studies, although associations were not consistently found across all studies located and the total number of studies to date is small. 

Findings and conclusions for LBW differ with conclusions of Hohmann’s review [16] because we have included one large population based cohort study [44] published after the Hohmann review, one large study from Japan [41] and one case control study from China [27] which were not included in that previous systematic review. These three studies gave supportive evidence for association between higher level of noise exposure and LBW.

*Small for gestational age* was investigated in three occupational studies [25,28,33] and noise didn’t reach statistical significance alone, but only in combination with other occupational factors like shift work. A large epidemiological study published in 2014 that used noise modeling software found that road traffic noise exposure was associated with SGA [44].

*Gestation length* was investigated in one occupational study [23] and one epidemiological study [41], but neither found significant risk for noise exposure. 

*Preterm birth* was investigated in five occupational studies [24,28,29,32,35] and only one study found an increased risk. This outcome was investigated in two epidemiological studies, one of which found significant associations for aircraft noise exposure above 85 dBA [41], while a second did not find an association with transportation noise (with lower exposure levels) [44].

*Spontaneous abortion* was investigated in three studies: one occupational study with subjective evaluation of noise exposure, which found significant risk in women that work in sales, service and office work [22], in another study was found significant risk in exposed women on the noise level above 95 dBA [27]. One study for environmental noise exposure over 6 hours daily (subjective evaluation) had found risk for recurrent spontaneous abortion [39].

*Threatened abortion* was associated with shift work and noise exposure in one occupational study [25], but another occupational study didn’t show significant risk [28].

*Congenital anomalies* were investigated in two studies [10,26], one occupational and one epidemiological, but neither study found significant associations.

Taken together, the small number of available studies were generally supportive of an association between noise exposure and adverse effects on birthweight, but publication bias cannot be ruled out and some studies of the studies had limitations in design. There was a very small number of studies on other reproductive outcomes and no clear suggestion of adverse associations other than for SGA.

### 3.4. The Biological Mechanism Underlying Influence of Noise Exposure on Reproductive Outcomes

New multi-disciplinary research on brain–body interactions triggered by stress in early pregnancy has shown that maternal biological responses, including localized inflammation in uterine tissue and sustained depression of progesterone production, challenge the endocrine-immune steady state during pregnancy, leading to serious consequences for the fetal environment. Recent basic science findings and new theoretical development around a ‘pregnancy stress syndrome’ associated with over-activation of the HPA axis warrant a new look at the epidemiological evidence around the age-old question of whether or not stress can actually cause human reproductive failure [6,7].

Noise exposure has been linked to increased levels of plasma catecholamines (norepinephrine and epinephrine) and this may be one of the mechanisms involved in observed effects noted above. In rats, norepinephrine infusion acutely reduces ovarian and uterine blood flow [13] and in guinea pigs, infusion of norepinephrine decreases placental blood flow by 24% to 46%, depending on the dose administered. Therefore, increased norepinephrine could cause decreases in blood flow that could adversely influence implantation and fetal health [14]. A mechanism by which the postimplantation stage might be affected by endogenously elevated maternal plasma CA levels is by reduction in blood flow through uterus. The noise exposed *vs*. control group plasma NE differences were less on the first day of exposure in both exposed groups and increased with exposure duration, indicating a cumulative effects [13]. CA synthesis is maintained at elevated levels during chronic restraint stress in rats [10,11,13]. 

Corticotropin-releasing hormone (CRH), the principal regulator of the HPA axis, has been identified in most female reproductive tissues including the uterus, the placenta, and the ovary [45]. Placental CRH has been proposed to directly modulate the endocrine function of placental trophoblasts, including the production of estrogen, ACTH, and prostaglandin, and is involved in the timing of parturition [45,46]. Remarkably the trajectory of CRH increase during pregnancy has been described to differ by ethnicity and also upon statistical adjusting for sociodemographic and biomedical factors [47]. Besides the regulatory function of CRH during pregnancy and parturition, a wealth of data indicates that high levels of glucocorticoids wield harmful effects on the uterus and fetus, and inhibit pituitary luteinizing hormone, and ovarian estrogen, and progesterone secretion. The notion of stress-triggered inhibition of progesterone secretion—or a more rapid metabolism—is supported by experimental evidence from animal studies. Here, exposure to stress in the form of restraint or sound induces abortion in pregnant mice via a significant reduction in progesterone levels, along with a reduce expression of progesterone receptor at the feto-maternal interface [48,49,50].

To summarize what is known currently about the human biological response to stress, the sympathetic nervous system is activated and NGF and SP are released and trigger an intense local inflammatory response, whereas a systemic inflammatory response generally may be suppressed. Accordingly it may be proposed that the immune response plays a sentinel role within the complex bodily response to stress, characterized on the one hand by the bias towards pro-inflammation/Th1 in response to SP, NGF and catecholamines, and on the other by the bias towards immunosuppression/Th2 in response to glucocorticoids.

## 4. Conclusions

Reproductive outcomes in humans result from complex interactions of individual physiological and psychological characteristics with demographic, ethnic factors, environmental and socioeconomic factors. Evidence considering the plausibility of an independent association between noise exposure and reproductive outcomes like LBW, preterm birth, spontaneous abortion, gestation length is sparse. However, the evidence available found to support these interactions.

The biological evidence points to contribution of noise exposure to reproductive failure in critical windows of gestational time via implantation failure, dysregulation of placentation, decrease of uterine blood flow. Recent evidence describes a hierarchy of biological mediators involved in a stress trigger to reproductive failure and a relatively new conceptual approach describes the stress susceptibility in mother and fetus via a pregnancy stress syndrome.

Epidemiological studies related to environmental or occupational noise exposure have shown that very high noise exposure on higher levels could be associated with low birthweight, but not with other investigated reproductive outcomes.

A major limitation of the studies investigated was the exposure assessment. As with other authors who have reviewed literature for noise related health outcomes, we would recommend inclusion of objective as well as subjective noise exposure assessments, assessment of time-activity patterns of subjects and use of noise propagation modeling. The numbers of studies identified was small and the methods were heterogeneous sо it is difficult to draw firm conclusions. Further research on the relation between noise exposure and reproductive outcomes is needed, given the ubiquitous exposure to different sources of noise and rising noise level in urban centers. LBW defined as birthweight < 2500 g and preterm birth defined as gestational age at birth less than 37 completed gestational weeks should be key outcomes in further prospective studies with emphasis on accurate and objective noise exposure assessment.

## 5. Recommendations

More research on associations between environmental noise exposures and reproductive outcomes is needed, using:

- objective and well-designed environmental noise exposure assessment
- well-designed epidemiological studies
- adjustment for confounding factors, such as life-style factors (smoking, alcohol use, drug use), characteristics of parents (parental weight and height, mother’s age, race, ethnicity, socioeconomic status *etc*. and gravidity and parity history for spontaneous abortion and congenital malformations
- adjustment for air pollution when considering outdoor transportation noise
- harmonized outcome definitions including use of birthweight < 2500 g for LBW preferably with information on gestational age and birth less than 37 completed gestational weeks for preterm birth, in order to obtain comparable results
